# Electrical, dielectric properties and study of AC electrical conduction mechanism of Li_0.9_□_0.1_NiV_0.5_P_0.5_O_4_

**DOI:** 10.1098/rsos.171472

**Published:** 2018-02-21

**Authors:** A. Rahal, S. Megdiche Borchani, K. Guidara, M. Megdiche

**Affiliations:** 1 Laboratory of Spectroscopic Characterization and Optical Materials (LaSCOM), University of Sfax, Faculty of Sciences, BP 1171, 3000 Sfax, Tunisia; 2 Higher Institute of Computer Science and Multimedia of Sfax (ISIMS), Technological Center of Sfax, BP 242, SakietEzzit, 3021 Sfax, Tunisia

**Keywords:** impedance spectroscopy, AC conductivity, non-overlapping small polaron tunnelling model

## Abstract

In this paper, we report the measurements of impedance spectroscopy for a new olivine-type lithium deficiency Li_0.9_□_0.1_NiV_0.5_P_0.5_O_4_ compound. It was synthesized by the conventional solid-state technique. All the X-ray diffraction peaks of the compound are indexed, and it is found that the sample is well crystallized in orthorhombic olivine structure belonging to the space group *Pnma*. Conductivity and dielectric analyses of the sample are carried out at different temperatures and frequencies using the complex impedance spectroscopy technique. The electrical conductivity of Li_0.9_□_0.1_NiV_0.5_P_0.5_O_4_ is higher than that of parent compound LiNiV_0.5_P_0.5_O_4_. Temperature dependence of the DC conductivity and modulus was found to obey the Arrhenius law. The obtained values of activation energy are different which confirms that transport in the title compound is not due to a simple hopping mechanism. To determine the conduction mechanism, the AC conductivity and its frequency exponent have been analysed in this work by a theoretical model based on quantum mechanical tunnelling: the non-overlapping small polaron tunnelling model.

## 1. Introduction

AC conductivity, DC conductivity and dielectric dispersion are characteristics of many ionic conductors. Analyses of dielectric properties are important to investigate the nature of barrier properties for applications in devices. Recently, olivine structure materials have been found to be very promising cathode materials for lithium-ion batteries [1]. The Li-ion battery has changed enormously our lifestyles. It is, nowadays, the power source of choice for portable electronic devices such as portable phones and laptops [2].

Besides, AC impedance spectroscopy is a powerful technique for the dielectric, electrical and modulus study of mixed electronic ionic conductors [3,4].

Olivine-type compounds are of great interest as candidate materials for lithium-ion batteries, ever since the discovery of the insertion–deinsertion properties of lithium in LiFePO_4_ [1]. This result has led to new investigations of lithium storage electrodes among materials belonging to families of polyanionic compounds, especially olivine-type compounds.

Olivine-type LiNiPO_4_ [5,6] has been considered as a most competitive positive electrode active material for lithium-ion batteries. The previously studied oxide LiNiV_0.5_P_0.5_O_4_ [7], which belongs to the olivine-type family, is also a promising cathode material for application in lithium-ion batteries. According to our knowledge, the lacunar plays an important role in the physical properties of many oxides. Therefore, there is no work done on the lacunar in the orthophospho-vanadium mixed oxide. From this, comes our idea of creating a vacancy in the lithium site of LiNiV_0.5_P_0.5_O_4_ compound, for the research of a new cathodic material.

In this present paper, we report here for the first time the electric, dielectric properties and the conduction mechanism of Li_0.9_□_0.1_NiV_0.5_P_0.5_O_4_ mixed oxide by means of impedance spectroscopy.

## 2. Experiment

The Li_0.9_□_0.1_NiV_0.5_P_0.5_O_4_ ceramics were prepared by conventional methods. Analytical grade reagents with 99% purity of N_2_H_9_PO_4_, Ni_3_CO_11_H_12_, Li_2_CO_3_ and V_2_O_5_ were used as raw materials with appropriate mass according to the stoichiometric ratio and were mixed in a mortar for 3 h. The mixture of these materials was progressively heated from room temperature to 573 K in a first step in order to eliminate NiCO_3_2Ni(OH)_2_ and H_2_O. This step allows the decomposition and release of gas according to the following reaction:

$$\frac{0.9}{2}{\text{Li}}_{2}\text{C}{\text{O}}_{3}+\frac{1}{3}\text{N}{\text{i}}_{3}\text{C}{\text{O}}_{11}{\text{H}}_{12}+\frac{\text{1}}{\text{4}}{\text{V}}_{2}{\text{O}}_{5}+\frac{\text{1}}{\text{2}}{\text{N}}_{2}{\text{H}}_{9}\text{P}{\text{O}}_{4}\Rightarrow \text{L}{\text{i}}_{0.9}{◻}_{0.1}\text{Ni}{\text{V}}_{0.5}{\text{P}}_{0.5}{\text{O}}_{4}.$$

The product obtained was weighed again and manually ground, and the powder was then pressed in the form of a pellet of thickness of approximately 1–1.1 mm and 8 mm in diameter to facilitate the evaporation mechanisms, condensation and diffusion at the reaction in the solid state. The pellets obtained are sintered at a temperature of 933 K for 10 h.

At room temperature, the sample was characterized by its X-ray powder pattern using a Phillips powder diffractometer PW1710 with CuK*α* radiation (*λ* = 1.5405 Å) in a wide range of Bragg angles (10° ≤ 2*θ* ≤ 80°).

The electrical measurements were performed using two platinum electrode configuration. The finely grain samples were pressed into pellets of 8 mm diameter and 1.1 mm thickness before being sandwiched between these electrodes. Moreover, the measurements were performed as a function of both temperature (590–670 K) and frequency (209 Hz–1 MHz) using a TEGAM 3550 ALF impedance analyser.

## 3. Results and discussion

### 3.1. Crystalline parameters

**Figure 1.**
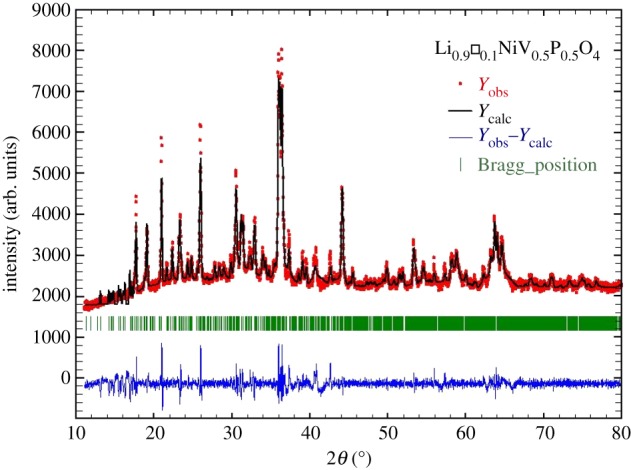
Powder X-ray diffraction pattern of Li_0.9_□_0.1_NiV_0.5_P_0.5_O_4_. Here, *Y*_obs_, *Y*_calc_, *Y*_obs_–*Y*_calc_ and Bragg position represent the experimental data, calculated data, the difference of experimental and calculated data and Bragg's positions, respectively.

The X-ray diffraction pattern of the Li_0.9_□_0.1_NiV_0.5_P_0.5_O_4_ compound at room temperature is shown in figure 1. The data were refined by the Rietveld technique using the Fullprof program. All the X-ray peaks were easily indexed in the orthorhombic olivine structure belonging to the space group *Pnma*, and the refined unit cell parameters are: *a* = 18.743 Å, *b* = 9.911 Å, *c* = 8.896 Å, *α* = *β* = *γ* = 90° and *V* = 1689.01 Å^3^. The structural results are in agreement with the previously reported results for the parent compound LiNiV_0.5_P_0.5_O_4_ [7].

### 3.2. Impedance studies

The interface, grain and grain boundary properties are studied using the complex impedance formalisms that include the determination of capacitance, relaxation frequency and ionic conductivity [8].

**Figure 2.**
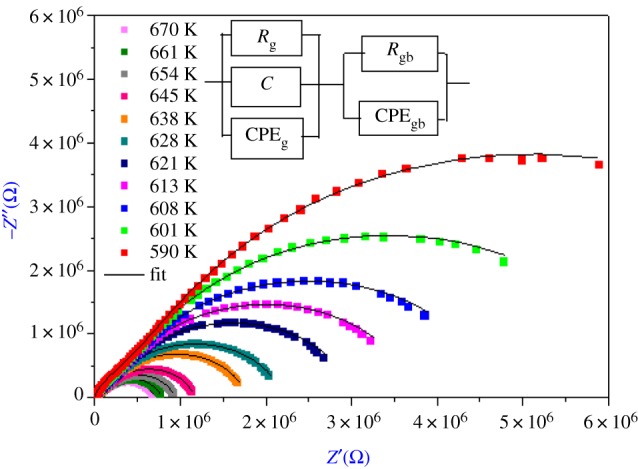
Complex impedance plot of Li_0.9_□_0.1_NiV_0.5_P_0.5_O_4_ at various temperatures with equivalent circuit.

The plot of −*Z*′′ versus *Z*′ (Nyquist diagram) at different temperatures (590–670 K) of Li_0.9_□_0.1_NiV_0.5_P_0.5_O_4_ sample is shown in figure 2. There are mainly two overlapping semicircles, which correspond to grain interiors [9] (the semicircle at low frequency) and grain boundaries (the semicircle at high frequency). In other words, centres of semicircles that compose the total electric response are centred below the real axis (*Z*′), which confirms the presence of non-Debye type of relaxation in the materials.

The impedance data have been fitted to an equivalent circuit (inset in figure 2) consisting of series combination of grain interior and grain boundary. First consists of parallel combination of resistance (*R*_g_), capacitance (*C*) and a constant phase element CPE_g_, and second consists of parallel combination of resistance (*R*_gb_) and a constant phase element CPE_gb_.

Impedance of the capacity of the fractal interface CPE is given by the following equation:

3.1 $$z_{\mathrm{CPE}}=\frac{1}{Q{\left(\;j\omega \right)}^{\alpha }},$$

where *Q* indicates the value of the capacitance of the CPE element and *α* is the degree of deviation with respect to the value of the pure capacitor.

The expressions, which relate the modulus |*Z*| and phase *θ* with frequency, are obtained from the real part (*Z*′) and imaginary part (*Z*′′) of the complex impedance of the above equivalent circuit as follows:

3.2 $$|Z|=\sqrt{Z^{\prime 2}+Z^{\prime \prime 2}}$$

and

3.3 $$\theta =\tan^{-1}\left(\frac{Z^{\prime \prime }}{Z^{\prime }}\right),$$

where

3.4 $$\begin{array}{rl} Z^{\prime } & =\frac{{R_{g}}^{-1}+Q_{g}\omega^{\alpha_{g}}\cos(\alpha_{g}\pi /2)}{{\left[{R_{g}}^{-1}+Q_{g}\omega^{\alpha_{g}}\cos(\alpha_{g}\pi /2)\right]}^{2}+{\left[C\omega^{\alpha_{g}}\sin(\alpha_{g}\pi /2)\right]}^{2}}\\ & \;+\frac{R_{\mathrm{gb}}+{R_{\mathrm{gb}}}^{2}Q_{\mathrm{gb}}\omega^{\alpha_{\mathrm{gb}}}\cos(\alpha_{\mathrm{gb}}\pi /2)}{{\left[1+R_{\mathrm{gb}}Q_{\mathrm{gb}}\omega^{\alpha_{\mathrm{gb}}}\cos(\alpha_{g}\pi /2)\right]}^{2}+{\left[R_{\mathrm{gb}}Q_{\mathrm{gb}}\omega^{\alpha_{\mathrm{gb}}}\sin(\alpha_{\mathrm{gb}}\pi /2)\right]}^{2}} \end{array}$$

and

3.5 $$\begin{array}{rl} -Z^{\prime \prime } & =\frac{C\omega^{\alpha_{g}}+Q_{g}\omega^{\alpha_{g}}\sin(\alpha_{g}\pi /2)}{{\left[{R_{g}}^{-1}+Q_{g}\omega^{\alpha_{g}}\cos(\alpha_{g}\pi /2)\right]}^{2}+{\left[C\omega^{\alpha_{g}}\sin(\alpha_{g}\pi /2)\right]}^{2}}\\ & \;+\frac{{R_{\mathrm{gb}}}^{2}Q_{\mathrm{gb}}\omega^{\alpha_{\mathrm{gb}}}\sin(\alpha_{\mathrm{gb}}\pi /2)}{{\left[1+R_{\mathrm{gb}}Q_{\mathrm{gb}}\omega^{\alpha_{\mathrm{gb}}}\cos(\alpha_{g}\pi /2)\right]}^{2}+{\left[R_{\mathrm{gb}}Q_{\mathrm{gb}}\omega^{\alpha_{\mathrm{gb}}}\sin(\alpha_{\mathrm{gb}}\pi /2)\right]}^{2}}. \end{array}$$

Thus, the presentation of the impedance data as a Bode plot gives information that helps to ascertain more directly the different conduction processes involved in the sample.

**Figure 3.**
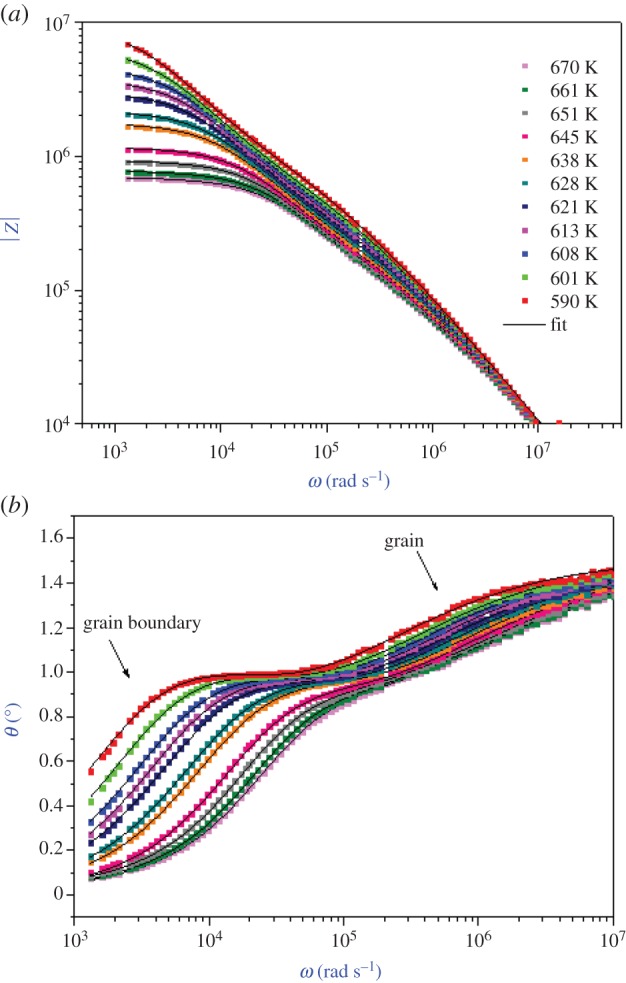
Bode plots for Li_0.9_□_0.1_NiV_0.5_P_0.5_O_4_: |*Z*| versus frequency (*a*) and phase *θ* versus frequency (*b*).

Bode plots of the Li_0.9_□_0.1_NiV_0.5_P_0.5_O_4_ sample are presented in figure 3*a*,*b*.

The good conformity of calculated lines with the experimental measurement in figures 2 and 3*a*,*b* indicates that the suggested equivalent circuit describes the crystal–electrolyte interface reasonably well. Fitted values of parameters (grain and grain boundary), determined using the Zview software for different temperatures, are listed in table 1. The capacitance values for the high- and the low-frequency semicircles are found to be in the range of pF and nF, respectively, proving that the observed semicircles represented the bulk and the grain boundary response of the system, respectively.

**Table 1.** Extracted parameters of the equivalent circuit.

*T* (K)	*R*_g_ (10^5^ Ω)	*C* (pF)	*Q*_g_ (10^−8^ F)	*α*_g_	*R*_gb_ (10^6^ Ω)	*Q*_gb_ (nF)	*α*_gb_
590	8.89	13.60	0.75	0.47	8.91	1.6	0.88
601	8.39	12.5	1.05	0.45	5.67	1.5	0.89
608	7.99	12.1	1.30	0.45	3.93	1.3	0.90
613	7.29	11.8	1.46	0.44	3.07	1.5	0.91
621	6.69	11.5	1.60	0.44	2.40	1.2	0.91
628	5.99	11.4	1.88	0.46	1.65	1.4	0.92
638	5.39	10.8	1.89	0.49	0.91	1.6	0.93
645	5.17	9.77	1.31	0.48	0.70	1.0	0.97
654	3.93	9.76	1.25	0.53	5.73	1.0	0.96
661	3.40	9.55	1.15	0.50	0.46	1.1	0.95
670	3.09	9.42	1.09	0.51	0.41	1.2	0.98

Relying on the bulk resistance values and the sample dimensions, the grain electrical conductivity, grain boundary conductivity and total conductivity can be calculated at each temperature using the following equations:

3.6 $$\begin{array}{rl} \sigma_{g} & =\frac{e}{S\times R_{g}} \end{array}$$

3.7 $$\begin{array}{rl} \sigma_{\mathrm{gb}} & =\frac{e}{R_{\mathrm{gb}}\times S}\times \frac{C_{g}}{C_{\mathrm{gb}}} \end{array}$$

3.8 $$\begin{array}{rl} \;\text{and}\;\sigma_{\mathrm{tot}} & =\frac{e}{(R_{g}+R_{\mathrm{gb}})\times S}=\frac{e}{R_{\mathrm{tot}}\times S} \end{array}$$

where *e* and *S* are, respectively, the thickness and the area of the pellet, and the pure capacitance *C* can be obtained using the following equation:

3.9 $$C_{\mathrm{gb}}={R_{\mathrm{gb}}}^{\left(1-\alpha_{\mathrm{gb}}/\alpha_{{\;}_{\mathrm{gb}}}\right)}\times {Q_{\mathrm{gb}}}^{1/\alpha_{\mathrm{gb}}}.$$

**Figure 4.**
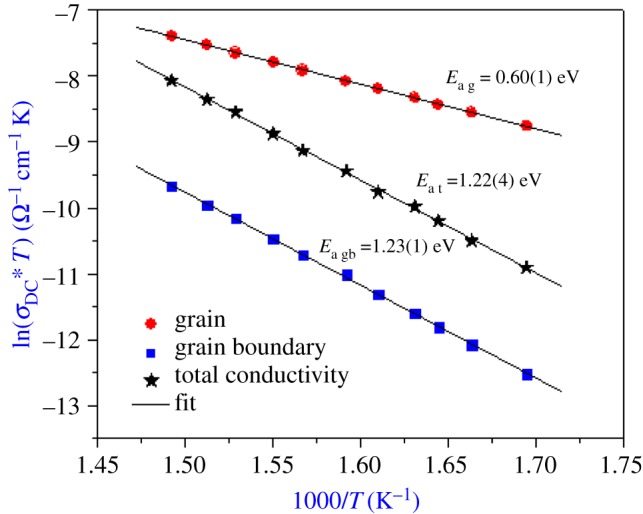
Variation of ln (σ*_DC _ *T*) versus (1000/*T*).

The temperature dependence of the grain electrical conductivity (*σ*_g_), grain boundary conductivity (*σ*_gb_) and total conductivity (*σ*_tot_) is shown in figure 4. The linearity of ln(σ*_DC _ *T*) versus 1000/*T* shows that this material does not present any phase transition in the studied temperature range. All the *σ*_g_, *σ*_gb_ and *σ*_tot_ conductivities increase linearly with temperature, which indicates that the electrical conduction in the sample is a thermally activated transport process and obeys the Arrhenius law [10]: *σ*_DC_*T* = *σ*_0_exp (−*E*_a_/*k*_B_*T*), where *σ*_0_ is the pre-exponential factor, *k*_B_ is the Boltzmann constant and *E*_a_ is the energy of activation of conduction.

The values of the activation energy, estimated from the Arrhenius plots of *σ*_g_, *σ*_gb_ and *σ*_tot_ of the sample, are, respectively, *E*_ag_ = 0.60(1) eV, *E*_agb_ = 1.23(1) eV and *E*_at_ = 1.22(4) eV. Those of the pre-exponential factor *σ*_0_ are, respectively, of the order: *σ*_0 g _= 1.45 × 10^1^ Ω^−1^ cm^−1 ^K, *σ*_0gb _= 8.59 × 10^4^ Ω^−1^ cm^−1^ K and *σ*_0t _= 4.16 × 10^5^ Ω^−1^ cm^−1^ K.

According to the Arrhenius plots, we note that the activation energy changes with lithium deficiency in Li_1−*x*_□*_x_*NiV_0.5_P_0.5_O_4_ (*x* = 0 and 0.1) (table 2). Its value for the parent compound is lower than for *x* = 0.1 [7]. In fact, the volume of the unit cells increases; the cations responsible for conduction are much more easily released, thus requiring lower energy for their mobility. The value of the conductivity is also found to be dependent on the lithium deficiency (*x*). It exhibits an increase with the increase of vacancy content, so the lacunar in Li site has an important effect on the conductivity in Li_1−*x*_□*_x_*NiV_0.5_P_0.5_O_4_ mixed oxides.

**Table 2.** Space group, volume, activation energies and DC conductivities at 638 K for Li_0.9_□_0.1_NiV_0.5_P_0.5_O_4_ and LiNiV_0.5_P_0.5_O_4_ compounds.

mixed oxide	LiNiV_0.5_P_0.5_O_4_ [7]	Li_0.9_□_0.1_NiV_0,5_P_0,5_O_4_ this work
space group	*Pnma*	*Pnma*
*Z* formula units	4	4
volume (Å^3^)	1662	1689.01
*E*_ag_ (eV)	0.652	0.601
*σ*_DC_ (*T* = 638 K) (Ω^−1 ^cm^−1^)	1.04 × 10^−7^	1.38 × 10^−7^

### 3.3. Modulus studies

The complex electrical modulus M* formalism has been used in the analysis of the electrical properties because it gives the main information about the sample bulk. The real part (*M*′) and the imaginary one (*M*′′) of the complex modulus M * (M * (*ω*) = *M*′ + *j M*′′) have been obtained from the complex impedance data (Z * (*ω*) = *Z*′ − *j Z*′′) by the following relations:

$$M^{\prime }=\omega C_{0}Z^{\prime \prime }\;\text{and}\;M^{\prime \prime }=\omega C_{0}Z^{\prime }.$$

**Figure 5.**
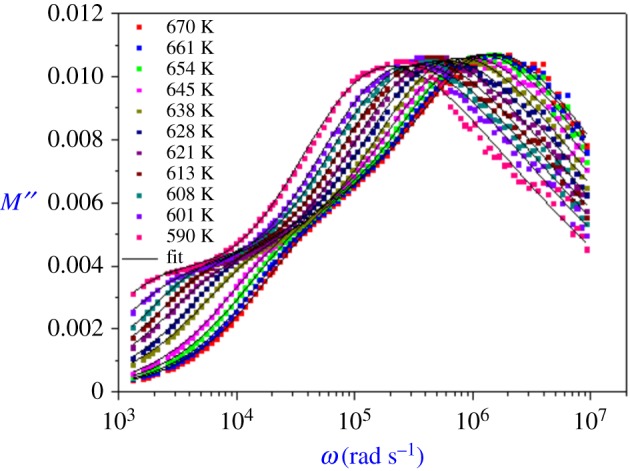
Variation of imaginary part of modulus *M*′′ with angular frequency for Li_0.9_□_0.1_NiV_0.5_P_0.5_O_4_ at different temperatures.

The variation of the frequency dependence of *M*′′ (*ω*) is shown in figure 5. It shows two distinct regions which are temperature dependent. *M*′′ shows a slightly asymmetric peak at each temperature. At lower temperature, two peaks are observed in these curves: at low frequency, the peak corresponds to the relaxation of the grain boundary and at higher frequency, it corresponds to the grain interior. Moreover, when temperature increases, modulus peak maxima shift to higher frequencies.

The imaginary part of the electric modulus has been fitted for different temperatures with an approximate frequency representation of the Kohlrausch–Williams–Watts function, proposed by Bergman [11]:

3.10 $$\begin{array}{rl} M^{\prime \prime }(\omega ) & =\frac{{M^{\prime \prime }}_{1\max}}{\left(1-\beta_{1})+(\beta_{1}/(1+\beta_{1}))[\beta_{1}{\left({\omega_{1}}_{\max}/\omega +\omega /\omega_{1\max}\right)}^{\beta_{1}}\right]}\\ & \;+\frac{{M^{\prime \prime }}_{2\max}}{\left(1-\beta_{2})+(\beta_{2}/(1+\beta_{2}))[\beta_{2}{\left({\omega_{2}}_{\max}/\omega +\omega /\omega_{2\max}\right)}^{\beta_{2}}\right]}. \end{array}$$

where *M*′′_max_ and *ω*_max_ are the peak maximum and the peak angular frequency of the imaginary part of the modulus, respectively. The value of *β* is positioned in the (0–1) range, which reflects the importance of coupling between mobile ions in the conduction process.

The extracted parameters by fitting the *M*′′ (*ω*) (figure 5) using the Bergman equation are shown in table 3.

**Table 3.** Parameters used for modulus fitting.

*T* (K)	*M*′′_1max_	*β*_1_	*ω*_1max_ (rad s^−1^)	*M*′′_2max_	*β*_2_	*ω*_2max_ (rad s^−1^)
670	0.0044	0.59231	69073.8129	0.00926	0.49241	2136920.543
661	0.00412	0.63376	53549.2211	0.0096	0.47841	1832609.313
654	0.00394	0.64769	41367.0362	0.00974	0.46968	1546937.422
645	0.00359	0.68906	28116.0523	0.00993	0.45511	1189080.492
643	0.00339	0.66826	17575.4988	0.01001	0.45685	939504.7124
628	0.00328	0.71888	12788.4282	0.01006	0.44497	733530.8376
621	0.00318	0.66849	9145.578	0.01002	0.44828	597481.3751
613	0.00308	0.71279	6839.38362	0.01008	0.44457	475134.0426
608	0.00305	0.73353	5214.41416	0.01006	0.43747	388462.0478
601	0.00296	0.67343	3670.62275	0.00999	0.44146	324029.9456
590	0.00296	0.70835	2322.21233	0.01004	0.44003	224002.2343

The most probable relaxation time follows the Arrhenius law:

3.11 $$\omega_{\max}=\omega_{0}\exp^{\left(-E_{a}/k_{B}T\right)},$$

where *E*_a_ is the activation energy and *ω*_0_ is the pre-exponential factor.

**Figure 6.**
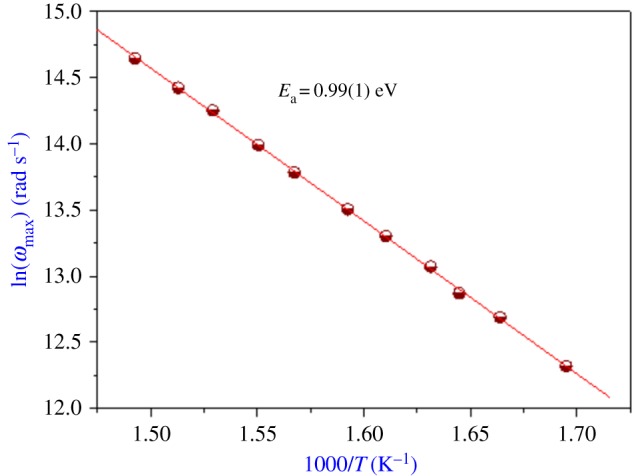
The temperature dependence of the conductivity relaxation frequency for grains.

The temperature dependence of the grain relaxation frequency is shown in figure 6. This variation obeys to the Arrhenius behaviour with activation energy equal to 0.99(1) eV. This obtained value (from modulus analysis) is different from that of the impedance measurement study (0.60(1) eV). We conclude that the ion transport is not due to the hopping mechanism.

### 3.4. Dielectric studies

Dielectric relaxation studies are important to understand the nature and the origin of dielectric losses, which may be useful in determining the structure and defects in solids.

The dielectric relaxation is described by a Cole–Cole model, which gives the frequency-dependent complex permittivity in the form [12,13]

3.12 $$\varepsilon^{*}(\omega )=\varepsilon_{\infty }+\frac{\varepsilon_{s}-\varepsilon_{\infty }}{1+{\left(j(\omega /\omega_{1})\right)}^{1-\alpha }}+\frac{\sigma_{0}}{j\varepsilon_{0}\omega },$$

where *σ*_0_ is specific conductivity, *ε*_∞_ is dielectric constant at infinite frequency and *ϵ*_s_ is static dielectric constant.

The imaginary part of ε* is [14]

3.13 $$\varepsilon^{\prime \prime }(\omega )=\frac{\left(\varepsilon_{s}-\varepsilon_{\infty }){\left(\omega /\omega_{1}\right)}^{1-\alpha }\sin((1-\alpha )\pi /2\right)}{1+2{\left(\omega /\omega_{1}\right)}^{1-\alpha }\cos(1-\alpha )\pi /2+{\left(\omega /\omega_{1}\right)}^{2(1-\alpha )}}+\frac{\sigma_{0}}{\varepsilon_{0}\omega }.$$

**Figure 7.**
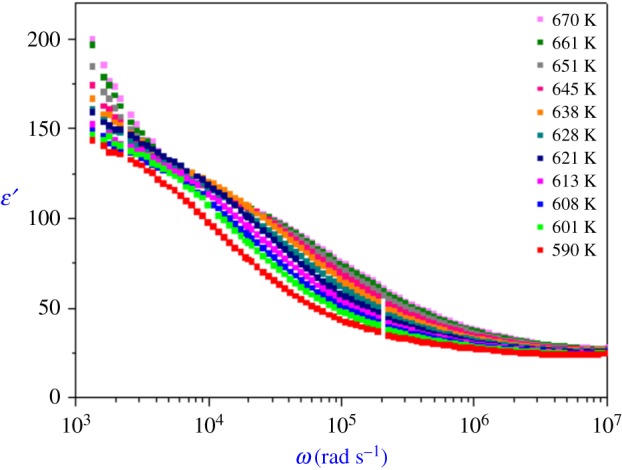
Variation of dielectric constant *ϵ*′ versus frequency at various temperatures.

**Figure 8.**
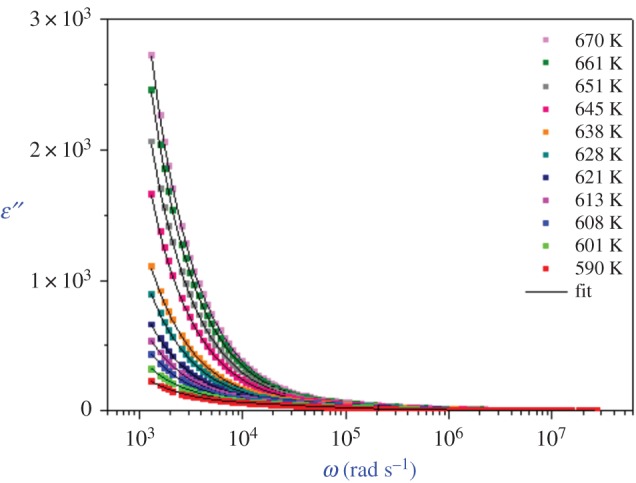
Variation of dielectric loss *ϵ*′′ versus frequency at various temperatures.

The angular frequency-dependence plots of the real and imaginary parts, *ε*′ and *ε*′′, of the complex dielectric permittivity (ε*) are shown in figures 7 and 8, respectively. At lower frequencies, the *ε*′ values increase with decreasing frequency with a rapid rise at high temperatures. In the intermediate frequency range, it shows a plateau. The rise of *ε*′ is due to the sample electrode interface polarization.

The frequency dependence of *ε′′* shows that two straight lines with different slopes separated by the frequency region are present in the frequency dependence of *ε*′′. This linear behaviour at both the high and low frequencies shows that there are definitely two processes contributing to the conduction of the sample: intra- and intergranular conductions.

### 3.5. AC conductivity

The AC conductivity has been calculated from the real and the imaginary parts of the impedance data measured over a study range of temperatures using the relation [15]

3.14 $$\sigma_{\mathrm{AC}}(\omega )=\frac{e}{S}\times \frac{Z^{\prime }}{Z^{\prime 2}+Z^{\prime \prime 2}}.$$

**Figure 9.**
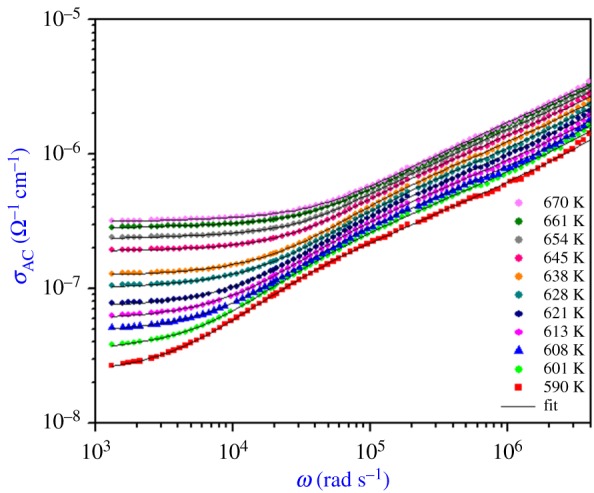
Frequency dependence of AC conductivity at various temperatures of Li_0.9_□_0.1_NiV_0.5_P_0.5_O_4._

The frequency dependence of AC conductivity in Li_0.9_□_0.1_NiV_0.5_P_0.5_O_4_ at different temperatures is shown in figure 9. It is clear from the plot that the range of low-frequency conductivity plateau increases with temperature, while the temperature dependency is less prominent in the high-frequency region. On the other hand, the conductivity is found to be frequency independent in the low-frequency regime.

The phenomenon of the conductivity dispersion is analysed by the following equation:

3.15 $$\sigma_{\mathrm{AC}}(\omega )=\frac{\sigma_{s}}{1+\tau^{2}\omega^{2}}+\frac{\sigma_{\infty }\tau^{2}\omega^{2}}{1+\tau^{2}\omega^{2}}+A\omega^{s},$$

where *σ*_s_ is the conductivity at low frequencies, *σ*_∞_ is an estimate of conductivity at high frequencies, *ω* is the angular frequency, *τ* represents the characteristic relaxation time, *A* is a pre-factor that depends on the temperature and composition, and *s* (0 < *s* < 1) is the frequency exponent.

**Figure 10.**
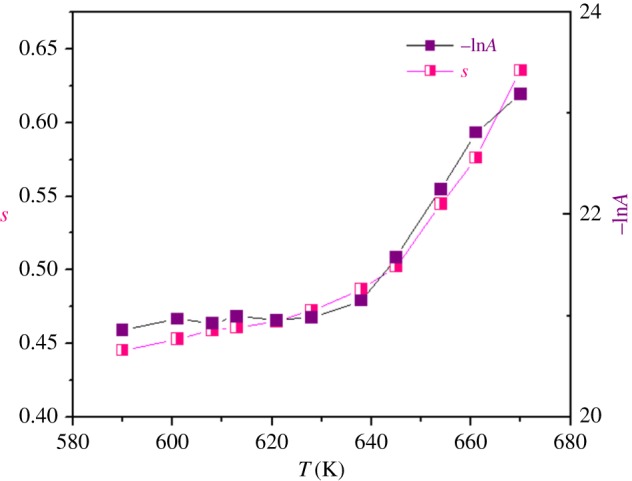
Variation of universal exponents *s* and *A* as a function of temperature.

*s* represents the degree of interaction between mobile ions with the environments surrounding them, and *A* determines the strength of polarizability. Equation (3.15) has been used to fit AC conductivity data. In the fitting procedure, *A* and *s* values vary simultaneously to get best fits as shown in figure 10.

**Figure 11.**
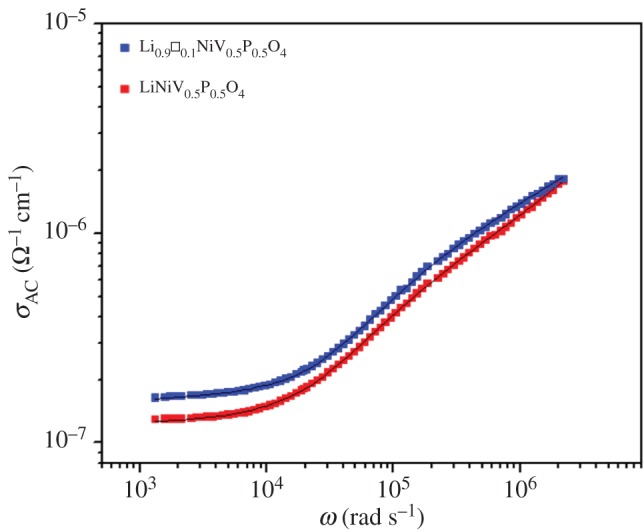
Frequency dependence of AC conductivity at 638 K of Li_1−*x*_□*_x_*NiV_0.5_P_0.5_O_4_ (*x* = 0 and 0.1)_._

For comparison, we have plotted in figure 11 *σ*_AC_(*ω*) for Li_1−*x*_□*_x_*NiV_0.5_P_0.5_O_4_ (*x* = 0 and 0.1) mixed oxides at 638 K. We can deduce that the electrical conductivity of the vacancy compound Li_0.9_□_0.1_NiV_0.5_P_0.5_O_4_ is higher than that of the parent one LiNiV_0.5_P_0.5_O_4_.

### 3.6. Theory investigation of non-overlapping small polaron tunnelling conduction mechanism

The behaviour of the frequency exponent as a function of temperature can be used to determine the origin of conduction mechanism. In the literature, several models [16–19], such as the non-overlapping small polaron tunnelling (NSPT) model, the quantum mechanical tunnelling model, the overlapping large-polaron tunnelling model and correlated barrier hopping (CBH) model, have been proposed to investigate the conduction mechanism based on the variation of frequency exponent with temperature and frequency.

For the presently studied Li_0.9_□_0.1_NiV_0.5_P_0.5_O_4_ sample, frequency dependence of *s* has increasing trends with temperature, suggesting the NSPT model (figure 10).

Regarding the values of *s* and its variation versus temperature, we conclude that *s* remains less than unity. Consequently, the NSPT seems to be the most interesting model related to the obtained results.

According to the tunnelling model, frequency exponent becomes

3.16 $$s=1+\frac{4k_{B}T}{W_{M}-k_{B}T\text{Ln}(\omega \tau_{0})},$$

where *W*_M_ is the polaron hopping energy, *k*_B_ the Boltzmann constant and *τ*_0_ is a characteristic relaxation time which is of the order of *τ*_0_ = 10^−13^ s.

According to this model, the AC conductivity is given by the following equation [20]:

3.17 $$\sigma_{\mathrm{AC}}=\frac{{\left(\pi e\right)}^{2}k_{B}T\alpha^{-1}\omega {\left[N(E_{F})\right]}^{2}{R_{\omega }}^{4}}{12},$$

where

3.18 $$R_{\omega }=\frac{1}{2\alpha }\left[\text{Ln}\left(\frac{1}{\omega \tau_{0}}\right)-\frac{W_{m}}{k_{B}T}\right],$$

where *α*^−1^ is the spatial extension of the polaron, *N*(*E*_F_) is the density of states near the Fermi level and *R_ω_* is the tunnelling distance.

**Figure 12.**
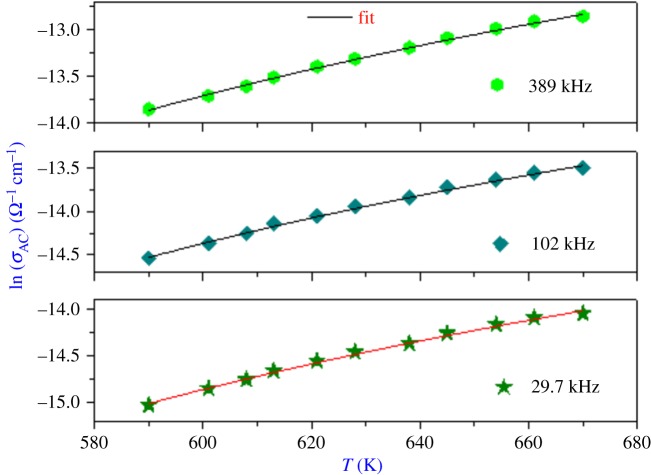
Temperature dependences of *σ*_AC_ at different frequencies of Li_0.9_□_0.1_NiV_0.5_P_0.5_O_4._

**Figure 13.**
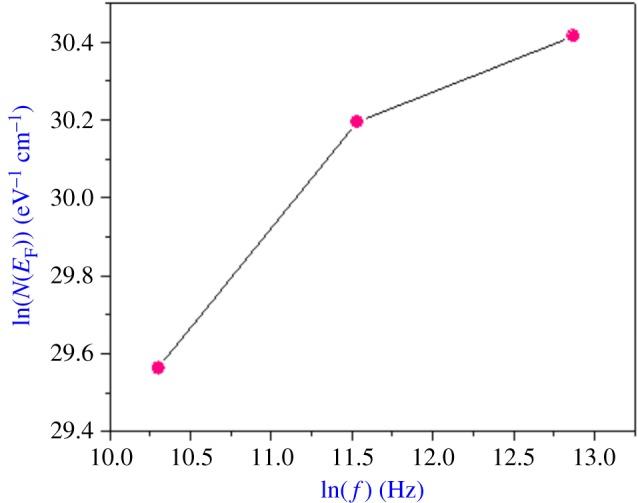
Variation of the parameter *N*(*E*_F_) (eV^−1 ^cm^−1^) according to the frequency (NSPT model).

The variation of the AC conductivity (ln(*σ*_AC_)) as a function of temperature at different frequencies is given in figure 12, which affirms the good conformity between the theoretical calculations and experimental data. The plot variations of the state density *N*(*E*_F_) versus frequency are shown in figure 13. It is clear that these parameters increase with frequency, which is in good agreement with the literature [21].

**Figure 14.**
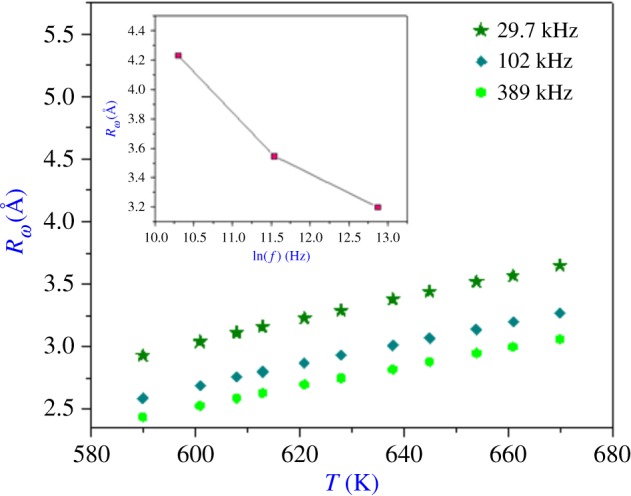
The temperature dependence of *R*_ω_ (Å) of Li_0.9_□_0.1_NiV_0.5_P_0.5_O_4_ at different indicated frequencies. The inset is its variation with frequency.

Among the results of refinement, we represent in figure 14 the variation of *R_ω_* with temperature at different frequencies (inset: the variation of *R_ω_* as a function of the frequency). It can be seen that in the NSPT model, the tunnelling distance (*R_ω_*) is of the order of the interatomic spacing [7] and its value decreases more rapidly with the increase in frequency.

## 4. Conclusion

In this work, we have synthesized for the first time an olivine type of Li_0.9_□_0.1_NiV_0.5_P_0.5_O_4_ compound by the solid-state reaction method. The lattice parameter for our lithium deficiency mixed oxide is slightly larger than that of the parent compound LiNiV_0.5_P_0.5_O_4_. It results in the vacancy sample favouring the expansion of the lattice volume and providing more space for lithium-ion transportation. Impedance plots show two semicircles, which confirm the presence of two relaxation processes associated with grain interior and grain boundary. The values of the activation energy in bulk of impedance (0.60(1) eV) and modulus (0.99(1) eV) spectrum suggest that the transport of ions in the present system is not by a hopping mechanism. Temperature dependence of the exponent suggests that the NSPT model can explain the conduction of the charge transport (Li^+^) in the ceramic compound Li_0.9_□_0.1_NiV_0.5_P_0.5_O_4_.

These results indicated that the Li_0.9_□_0.1_NiV_0.5_P_0.5_O_4_ is a promising cathode material for application in lithium-ion batteries.

## Data accessibility

This article has no additional data.
